# Bringing malaria diagnosis and treatment closer to the people: economic rationale for expanding malaria community case management to all ages in a rural district in Madagascar

**DOI:** 10.1186/s12936-025-05381-y

**Published:** 2025-05-04

**Authors:** Walter Ochieng, Julie R. Gutman, Catherine Dentinger, Aina Harimanana, Judickaelle Irinantenaina, Hobisoa Léa Razanadranaivo, Oméga Raobela, Aline Mukerabirori, Laurent Kapesa, Andres Garchitorena, Laura Steinhardt

**Affiliations:** 1 Global Health Center, US Centers for Disease Control and Prevention, Atlanta, GA USA; 2 National Center for Emerging and Zoonotic Infectious Diseases, US Centers for Disease Control and Prevention, Atlanta, GA USA; 3 U.S. President’s Malaria Initiative, US Centers for Disease Control and Prevention, Atlanta, GA USA; 4 Unité d’épidémiologie et de Recherche Clinique, Institut Pasteur de Madagascar, Antananarivo, Madagascar; 5 Programme National de Lutte Contre le Paludisme, Ministére de la Santé Publique de Madagascar, Antananarivo, Madagascar; 6 Management Sciences for Health, Antananarivo, Madagascar; 7 U.S. President’s Malaria Initiative, USAID, Antananarivo, Madagascar

**Keywords:** Malaria, Community case management, Madagascar, Economics

## Abstract

**Background:**

Expanding malaria community case management (mCCM) to all ages could shift the point-of-care to the community leading to improved healthcare access in underserved populations. This study assesses the economic viability of such an expansion in Farafangana district, Madagascar.

**Methods:**

A cluster-randomized trial was conducted across 30 health centres and the 502 community health workers (CHW) in their catchment areas, with the intervention arm implementing the age-expanded mCCM intervention. CHWs across both arms received training, supplies, and supervision to manage malaria. An economic evaluation assessed cost-effectiveness from health sector and societal perspectives, measuring outcomes in disability-adjusted life years (DALYs) averted. The impact of CHW compensation and economic risks were evaluated using sensitivity analyses.

**Results:**

Without CHW compensation, annual costs were $794,000, primarily for antimalarials and diagnostic tests. Incremental cost-effectiveness ratios (ICERs) per DALY averted ranged from -$21.86 to $212.42. From a societal perspective, the ICER was -$135.64, and -$243.29 including mortality benefits, meaning the intervention was cost-saving. The programme could avert 99.6 deaths and 3,721.7 DALYs annually, yielding $1,172,283 in net economic benefits. Sensitivity analyses supported these findings.

**Conclusions:**

Age-expanded mCCM is highly cost-effective and can enhance malaria treatment access in resource-limited settings.

**Supplementary Information:**

The online version contains supplementary material available at 10.1186/s12936-025-05381-y.

## Background

Malaria remains a leading cause of morbidity and mortality across sub-Saharan Africa (SSA), placing a tremendous burden on overstretched health systems and impeding economic development [1]. Despite intensive control efforts, progress in reducing malaria's impact has plateaued [2]. Innovative strategies are urgently needed to improve access to timely diagnosis and treatment for malaria, especially in remote areas with limited healthcare infrastructure [3].

Integrated community case management (iCCM) is an established intervention aimed at reducing child mortality through training and equipping community health workers to diagnose and treat common illnesses like malaria and diarrhoea [4, 5]. However, iCCM is restricted to children under five (CU5), leaving a significant portion of the population without access to community-based care [4]. Many countries are exploring expanding malaria community case management (mCCM) to all ages; however, the economic benefits of this strategy remain unclear [6].

The success of community health programmes hinges on a dedicated and motivated CHW workforce. While there is robust demand for CHW services across SSA, supply is constrained by inadequate recruitment, training, and retention. Insufficient incentives increase the opportunity cost of pursuing CHW roles, potentially deterring workforce entry/retention. Some CHWs may be intrinsically motivated by altruism and desire to serve their communities, which could partially offset absent financial compensation [7]. Nevertheless, over-reliance on intrinsic motivation is unsustainable [8] and can lead to burnout and attrition, hampering service delivery and quality; this risk may be aggravated by increasing workloads arising from expanded responsibilities. Appropriate extrinsic incentives, both monetary and non-monetary, are necessary to ensure a stable and motivated CHW workforce [7].

Many countries in SSA have struggled to transition towards a paid CHW workforce, given the high number of CHWs per population and many challenges to health systems financing. For example, Madagascar has one of the lowest per capita health investments in the world and relies on a network of over 35,000 unpaid CHWs to provide primary care services [9]. CHWs in Madagascar provide curative services for common childhood illnesses, support maternal and neonatal health, conduct health education, assist with vaccination campaigns, and patient referrals; on average they provide 1–2 consultations per day [6].

An analysis of the costs, health, and economic outcomes associated with age-expanded mCCM compared to standard iCCM were assessed in a randomized controlled trial [6]. Potential impacts of different CHW remuneration rates on the cost-effectiveness of age-expanded mCCM and the potential budgetary impacts of remuneration including affordability were also assessed. Value of information methods were used to check the potential value of further research. These findings can guide policy decisions on optimal healthcare resource allocation in Madagascar and inform malaria control programmes across SSA.

## Methods

A two-arm cluster-randomized trial involving 30 health facilities (15 intervention, 15 control) and the CHWs in their respective catchment areas was conducted in Farafangana, Madagascar from November 2019 to December 2021 [6]. All CHWs received a 2-day refresher training on mCCM and on the use of reporting tools, including patient registers and tally forms. The CHWs in the intervention arm also received additional training on malaria case management for older children and adults. All CHWs received necessary supplies including rapid diagnostic tests (RDTs) and artemisinin-based combination therapy (ACT), and enhanced supervision. In the control arm, only CU5 were treated by CHWs, while older patients were referred to health facilities. The primary outcome was the proportion of individuals two months or older with fever in the preceding fortnight who were tested and treated for malaria [6]. This economic evaluation was part of the trial.

### Data sources

This study utilized two main data sources: (1) routine monthly data from health facility and CHW registers from January 2019 to December 2021, including the number of consultations, patients with fever, RDTs done, RDT-confirmed malaria cases, and ACT delivered for each *fokontany* (village) and (2) primary and secondary cost data collected through the trial. Primary cost data were gathered through interviews with national malaria programme managers and implementing partners and their national and international pharmaceutical suppliers (Table S1). Secondary cost data were extracted from programme budgetary reports, published literature, and the World Health Organization Choosing Interventions that are Cost-Effective project (WHO-CHOICE) [10].

### Decision tree model

A decision tree model was used to categorize patients into those treated by CHWs versus those treated at health facilities –Supplementary Fig. S1. Two patient categories were assumed: those who would never seek care at health facilities and those who would have visited the facilities without the intervention. For the latter category, it was assumed that some patients would still choose facility services despite the intervention, influenced by factors such as proximity to the facility or perceived quality of care. Consequently, the intervention effects encompass costs associated with patients who would never have visited the health facilities (the economic extensive margin) and the savings from those who substitute treatment points to the less-resource-intensive CHW services (the economic intensive margin). Some costs are assumed for a proportion of facility services e.g., haemoglobin measurement, but are omitted for the CHW services.

### Model parameters

Treatment efficacy estimates, transition probabilities for progression to severe malaria and death, and probabilities of accessing inpatient care were extracted from published literature [11–15]. Disability weights associated with uncomplicated malaria, severe malaria, and related health sequelae were sourced from the Global Burden of Disease study 2019 [16]. Comprehensive sensitivity analyses were performed on key model parameters using the ranges delineated in Supplementary Table (ST) 1 to assess uncertainty in the results.

### Costs

Actual programme start-up costs, including national and district planning meetings; one-week trainings (hall hire, trainer costs, per diems, refreshments, printing, and transport); capital purchases (one motor vehicle and two motorcycles); and community sensitization were used. To calculate the annualized costs of the vehicles, a straight-line depreciation approach, spreading the purchase costs evenly across their expected useful lifetimes, was employed.

Health sector costs included additional malaria RDTs, ACT, consumables, training, community sensitization, enhanced supervision, capital investments, and overheads. The inpatient non-medical (hotel) cost estimates are derived from WHO-CHOICE estimates adjusted using a GDP deflator, and encompass personnel, capital, and operational costs, but not consumables or drugs [10]. From the societal perspective, productivity gains from early treatment, monetary value of severe cases and premature deaths averted, and averted out-of-pocket costs for patients and caregivers were included.

Productivity losses were estimated for caregivers and malaria patients aged 15 and above, in line with Madagascar's minimum working age labour laws. Caregiver time included accompanying minors to healthcare facilities, care at home, and time spent with patients admitted for severe malaria. Patient time costs were estimated from projected length of illness multiplied by the respective disability weights [17].

For CHWs, time costs were calculated based on their activities under the trial. It was assumed that CHWs travelled monthly to their catchment health facility for meetings and supplies and dedicated an hour weekly for administrative tasks like report preparation and drug inventory reconciliations. Given the relatively low patient volume, it was assumed that RDTs were performed serially, unlike batched testing at health facilities, resulting in higher time spent per individual patient [18, 19].

To estimate the economic value of time expended by CHWs, over-15 patients, and caregivers, an opportunity cost approach was employed, utilizing hourly agricultural (nominal) wage rates multiplied by time losses/benefits [20, 21]. This approach assumes that time spent on treatment and caregiving represents time lost from productive agricultural work, capturing the economic impact of malaria on households.

### Disability-adjusted life years (DALYs) averted

DALYs were quantified by estimating years of life lost due to premature death and years lived with disability, weighted by malaria severity [16]. The incremental cost-effectiveness ratios (ICER) for age-expanded versus standard mCCM were estimated by dividing incremental costs by DALYs averted. The 95% confidence intervals for the ICERs are estimated using a non-parametric bootstrap approach [22].

### Cost effectiveness analysis threshold (CET) and value of statistical life year (VSLY)

Cost-effectiveness (CE) has commonly been assessed using a threshold of one to three times the gross domestic product (GDP) per capita [23]. This approach has been criticized for being arbitrary, lacking theoretical basis, and not adequately filtering low value interventions [24, 25]. Instead, the health opportunity cost approach was used with an income elasticity parameter of 1.4, which adjusts how CE thresholds (CETs) scale with country GDP. In sensitivity analyses, this parameter was adjusted by 0.1 increments from 1.0 to 2.5 [25–27]. Results using the GDP-based method were compared with an $83[$26-$130] per DALY-averted threshold for Madagascar estimated by Pichon-Riviere et al*.* [28]. The value of statistical life year (VSLY) was estimated and then multiply it by discounted years of life lost to determine the economic costs of premature mortality [29].

### Discount rate and exchange rate

A discount rate of 3% was used in the base case scenario and 0% and 7% in sensitivity analyses. DALYs averted were discounted, but costs were not, as they were incurred within the year (except for vehicles). An exchange rate of 3905.4 Malagasy Ariary to 1 USD as of January 2022 was used [30].

### Sensitivity analyses, acceptability, affordability, and risk aversion

Employing univariate sensitivity analyses, the impact of varying individual parameters on ICERs were assessed, shown using tornado diagrams. Probabilistic sensitivity analyses (PSA) using 10,000 Monte Carlo simulations were done to assess joint parameter uncertainty and generate CE planes for individual ICERs [31]. Cost-effectiveness acceptability curves (CEAC) assessed the probability of age-expanded mCCM being cost-effective at different CETs [32, 33]. New interventions may impose significant costs on health systems and can be cost-effective yet unaffordable [34–36]. Cost-effectiveness affordability curves (CEAFC) with different budget constraints were used to check the probabilities of the intervention being both cost-effective and affordable [37] (Supplemental text and Fig. S2). To assess the need for future research to reduce parameter uncertainty, e.g., in severe cases averted, an expected value of perfect information (EVPI) analysis was conducted.

To account for decision-maker risk attitudes and explore the trade-off between cost and effectiveness under uncertainty, a cost-effectiveness risk-aversion curve (CERAC) analysis was conducted [38, 39]. For example, a 70% probability that an intervention is cost effective carries a non-insignificant risk that the intervention may not be so when implemented. Risk-averse decision-makers may therefore be hesitant to implement it. The CERAC adjusts for the risk that the intervention performs below a minimum acceptable threshold, i.e., downside risk. The CERACs were calculated for a range of CETs, depicting the relationship between these thresholds and the expected benefit-to-downside risk ratio for interventions below the threshold [38, 39]. The steps above were repeated by adding putative CHW monthly salaries using $1 increments from $1 to $150 to the costs. Data manipulation, analyses, and visualizations were performed using Python version 3.8 (Python Software Foundation, https://www.python.org/).

## Results

The estimated startup costs for Farafangana were $63,070 (Table 1), with trainings accounting for 59% and vehicles/motorcycles for 17%. The overall cost for one year of age-expanded mCCM in Farafangana was $794,000, with 91% of the costs spent on RDTs and antimalarials. The average RDT cost of $1.99 included assorted consumables (e.g., gloves and lancets), with the kit itself comprising 58% of the cost. It was estimated that each CHW will attend to an average of 294 additional patients per year (roughly one per day), resulting in an additional 111 h of work per year (Table 1 and Supplementary Tables 1 and 2).

**Table 1:** Key results from an analysis assessing costs and cost effectiveness of expanding malaria community case management to all ages in Farafangana, Madagascar from November 2019 to December 2021

Indicator	Mean	Range (95% CI)
Annual health facility consultations averted	200,275.1	[135,703.7, 285,122.9]
Annual hospitalizations averted	1699.2	[171.0, 6993.9]
Deaths averted per year	99.6	[60.9, 154.6]
Additional cases seen annually per CHW	294.3	[199.8–419.7]
Incremental CHW* hours per year	110.5	[72.0, 157.4]
Startup costs	$63,210.2	[$50,270.9, $77,918.8]
Admissions costs averted	$180,637.9	[$17,792.6, $756,770.1]
Clinic costs averted	$696,546.5	[$472,720.4, $990,598.4]
DALYs averted^#^	3,721.7	[2,054.2, 4,863.1]
Productivity benefits	$178,907.97	[$98,213.00, $305,963.64]
Caretaker benefits	$328,908.15	[$247,732.54, $439,707.53]
Averted deaths benefits (elasticity 1.4)	$344,014.63	[$210,648.39, $534,195.37]
Inpatient daily hotel costs	$6.51	[$4.75, $8.55]
CE^$^ threshold	$132.92	[$25.60, $690.2]
ICER^ health system	-$21.86	[-$136.93, $95.63]
ICER^ societal perspective (no deaths)	–$135.64	[-$1,918.45, 35.89]
ICER^ societal perspective (with deaths)	-$243.29	[-$2,056.15, -$49.31]

^*^CHW—community health worker

^#^DALY—disability adjusted life year

^$^CE—cost-effectiveness

^ICER—incremental cost-effectiveness ratio

Using the health opportunity cost approach [25] yielded a CET estimate of $133[95% CI: $26, $690] per DALY averted. This CET is significantly lower than the GDP-based $690 per DALY averted, or $345 per DALY averted based on 0.5 GDP per capita threshold [40]. However, it is slightly higher than previous estimates of $83[$26-$130] per DALY threshold for Madagascar [28]. Income elasticity thresholds above 1.5 to estimate the CET led to unrealistically low estimates. The value of a statistical life year of $1,328 was estimated for Madagascar using the baseline elasticity of 1.4 [26]. The baseline scenario was, therefore, a CET of $133 per DALY averted, a discount rate of 3%, and no CHW remuneration.

**Fig. 1:**
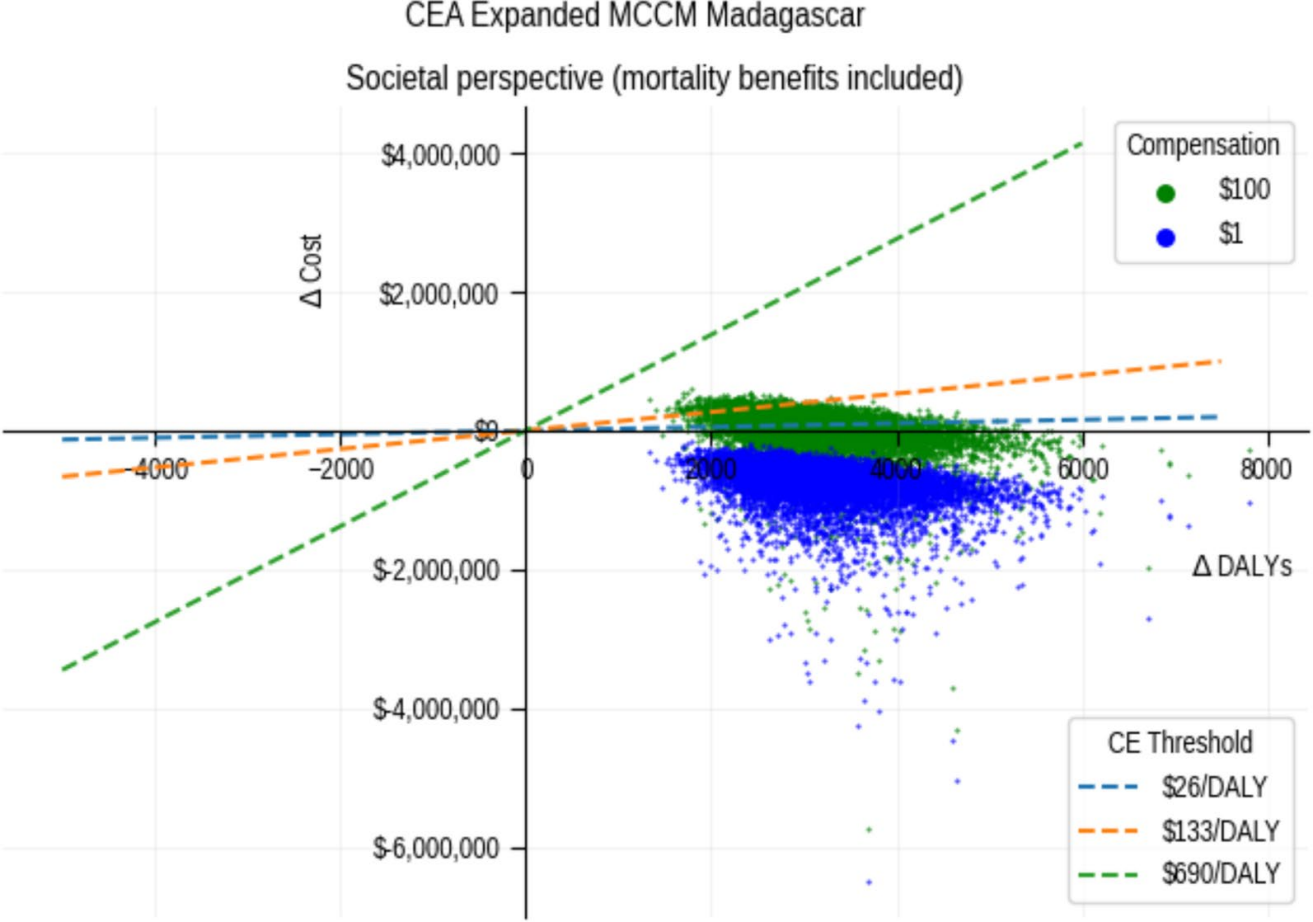
Cost-effectiveness analysis plane from a societal perspective comparing the impact of nominal compensation of $1 for CHWs versus $100 per month

Age-expanded mCCM was dominant—it cost less and yielded more benefit (hence negative ICERs). ICERs from the heath system perspective were –$22 [-$137, $99] (see Suppl. Fig. S3), indicating that inclusion of all ages costs less and yields more benefit than including CU5 only. From the societal viewpoint, the ICER was –$136[-$1,918, 36] without mortality benefits, and -$243 [-$2,056, -$49] per DALY averted if mortality benefits are included (see Figs. 1 and Suppl Fig. S3).

The choice of willingness to pay thresholds ($ per DALY averted) significantly impacted the probability that the intervention was cost-effective when using a compensation of $100 per month per CHW. All simulations assuming no compensation fall in the southeast quadrant—meaning the intervention is dominant.

**Fig. 2:**
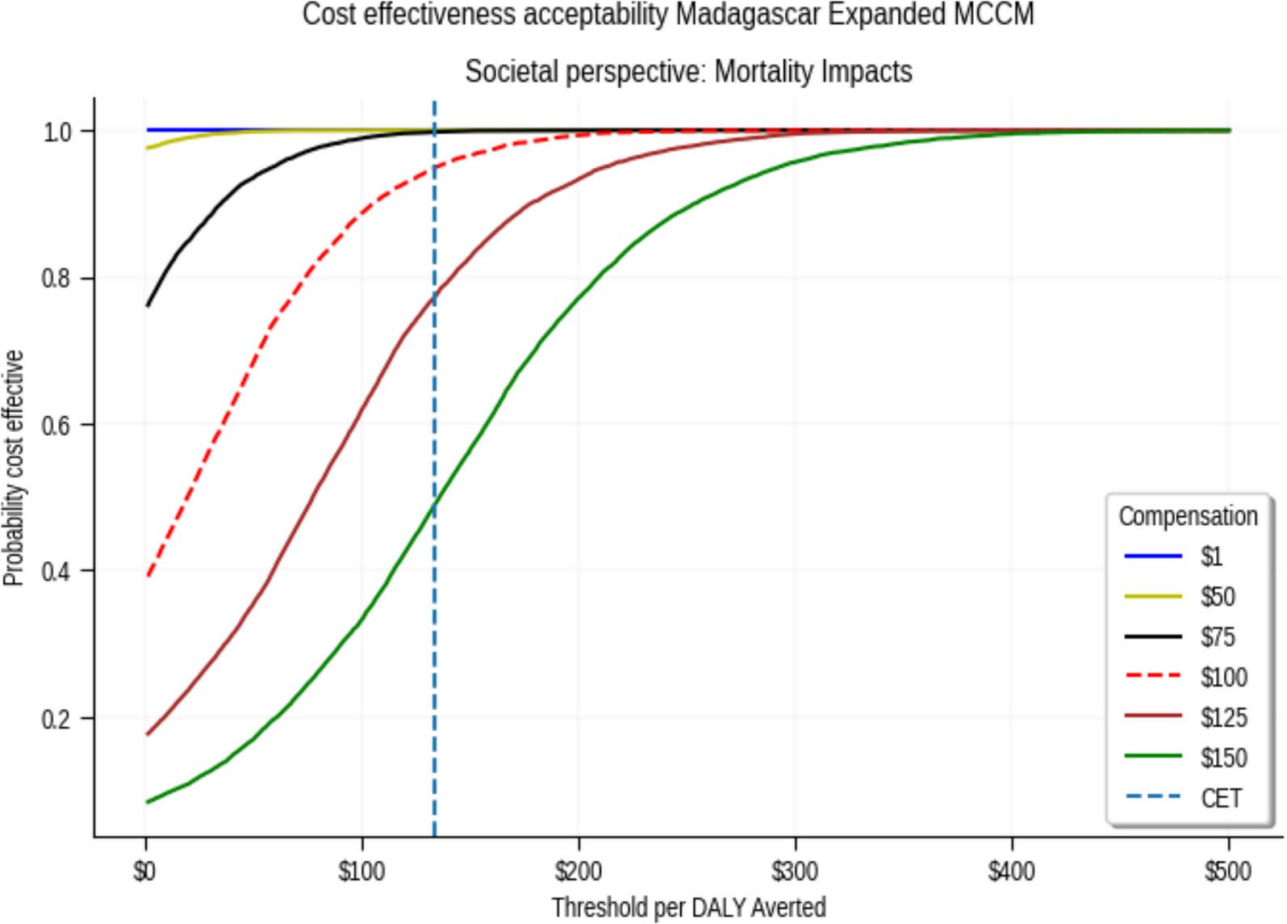
Cost effectiveness acceptability curve with willingness-to-pay thresholds on the x-axis. The vertical dashed line represents a threshold of $133 per DALY averted

There was greater than 80% probability that age-expanded mCCM was cost-effective with monthly CHW wages up to $45 from a health system perspective and up to $125 from a societal perspective (Fig. 2; Supplementary Tables 3 and 4).

**Fig. 3:**
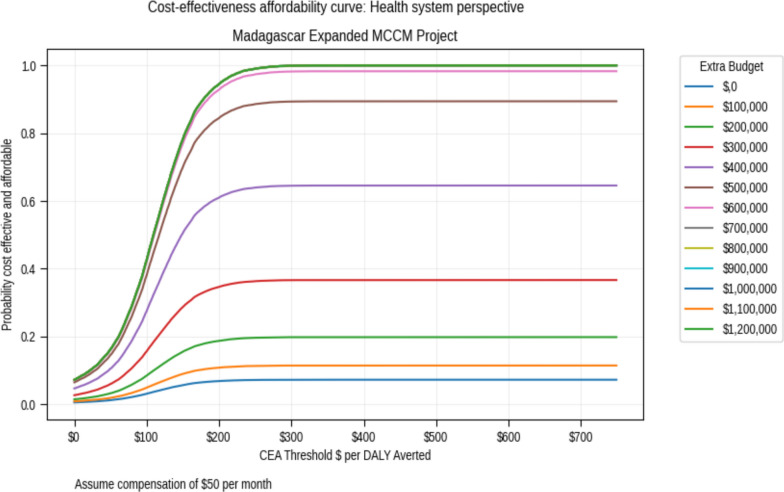
Cost-effectiveness affordability curve assuming a monthly salary of $50. Each curve represents the potential impact of a budgetary increase on affordability

Assuming no budgetary increase and no CHW remuneration, there is a 57% probability that the age-expanded mCCM is both cost-effective and affordable (feasible) from the health system perspective. A health sector budgetary increase of $100,000 raises this probability to 84% (Fig. 3). With monthly salaries of $50, the budgetary allocation to ensure that the intervention is feasible rises to $530,000.

In net monetary benefit terms, the intervention could save Farafangana District $496,849 [$166,437, $1,120,823] per year under the base scenario (Suppl Fig. S4). This rises to $827,980 from the societal perspective without averted deaths included and $1,172,284 with averted deaths included.

In the risk analysis using cost-effectiveness risk-aversion curve (CERACs) from a health system perspective, the net benefit-to-risk ratio is approximately 40 at a CET of $133 per DALY averted, and the intervention is virtually riskless above CETs of $185 per DALY averted (Suppl Fig. S5). The intervention is virtually riskless from a societal perspective. The health system perspective EVPI analysis yielded a value of $20.31, suggesting marginal potential economic benefits from further studies in this context.

### Sensitivity analyses

Sensitivity analyses revealed that, from the health system perspective, most uncertainty stemmed from assumptions regarding the number of admissions averted and facility costs saved.

Conversely, from the societal perspective, uncertainty primarily revolved around assumptions about income elasticity values, utility weights, and productivity days gained (Sppl Figs S6 and S7). In both perspectives, there was minimal uncertainty concerning drug and other consumable costs.

## Discussion

This study examined the economic ramifications of expanding mCCM to those over-5 years in a rural district in Madagascar. The intervention would cost the district health system approximately $794,000 annually, assuming CHWs are not paid, with drugs and RDTs accounting for over 91% of the costs. If CHWs were remunerated at $50 per month, the annual budget for the expanded project would rise to $1.2 million. It was estimated that the age-expanded mCCM programme would avert approximately 1,700 additional severe malaria cases annually, translating to an extra 100 deaths and 3,722 DALYs averted per year. The age-expanded mCCM was dominant (costing less and yielding more benefits) compared to status quo: -$22 per DALY averted from the health system perspective; -$136 the societal perspective without deaths; and -$243 from the societal perspective with deaths included.

These findings are highly sensitive to the choice of CETs, level of CHW compensation, and projections of deaths averted. For example, the intervention is cost effective under all scenarios assuming half the GDP-based CET [28, 41] of $345 per DALY averted and wages of $100 (Figs. 1 and 2). This declines to 59% if an estimated CET of $133 is used. Researchers conducting similar analyses should consider conducting sensitivity analyses around the choice of CETs [24, 25]. While the estimated CET was higher than $83, the intervention was still found to be cost effective under this threshold.

From the health system perspective, there is a 50% chance that the intervention is both effective and affordable if no additional funds are allocated to the project. This rises to 83% with a budgetary allocation of $100 K and no CHW remuneration. From the recommended societal perspective, the analyses suggest that the government could compensate CHWs between $50 and $140 per month while still achieving cost-effectiveness. This assumes that the government could transfer some of the economic benefits arising from increased productivity to CHW compensation.

A Mozambiquan study, while not looking at malaria, found that a $45 monthly CHW salary would increase the health budget by 362% but simultaneously increase by 56% the number of beneficiaries reached [42]. Taylor et al*.* found that it is unlikely for countries with GDP per capita under $1,200 to afford monthly CHWs of $80 without donor assistance [43]. It was estimated that paying all CHWs in Madagascar the minimum agricultural wage of $45 per month from the central budget would cost the country $19 million per year, or a per capita increase in health expenditure of $0.69. This translates to an increase of 13.8% of the national health salary budget, based on the 2022 health budget of $141 million [44]. It is unclear if Madagascar or donors have the fiscal space for this.

Investing in age-expanded mCCM has minimal risk from a health system perspective, especially with CETs above $186 (Suppl. Fig. S5). The intervention is virtually riskless from a societal perspective with the potential economic benefits substantially outweighing any residual uncertainties [37, 38]. The CERAC results alongside the EVPI ones that show minimal economic benefit of additional studies in the Farafangana context to obtain perfect data increase confidence that the intervention is worth implementing.

Direct patient productivity gains ($178,908) were less than productivity gains accrued through reduced caregiver time ($328,908). Productivity estimates are conservative because benefits stemming from earlier therapeutic intervention in those below the legal working age are omitted, though some are economically productive. It was assumed that there is an inverse relationship between age, number of malaria cases, and disease severity. Therefore, while the productivity impacts of younger individuals are ignored, their caretaking costs are still incorporated.

From the health system perspective, the primary sources of uncertainty stemmed from assumptions around the CETs, the number of severe malaria cases, and the number of health facility visits averted. Conversely, from the societal perspective, the results were most sensitive to assumptions around the CET, the number of severe malaria cases and deaths averted, and the choice of income elasticities used in estimating the value of statistical life years (Suppl Fig. S7). However, even under the most conservative assumptions, the intervention remained cost-effective.

This study has several limitations. The impact of compensation against incremental malaria cases seen and not against the range of services CHWs currently offer, e.g., treatment of diarrhoea and pneumonia among CU5 were considered. Therefore, the incremental impacts are conservative. GDP- deflated WHO CHOICE estimates from 2010 were used to estimate non-treatment out- and in-patient facility overheads. For instance, the weighted per patient outpatient visit cost, including personnel, utilities, capital, and equipment (but excluding drugs and other commodities) were estimated to be $1.49. This adjusted value may not reflect the current costs of these items [45]. Despite this, findings of this study align with other studies. For example, Hansen et al*.* [46] estimated the incremental costs of treating a child with malaria in a moderate to high transmission area at $3.00; using the same metric, this analysis estimates $3.06.

It was also assumed that the services will be offered seamlessly, with no gaps in service provision or supply chain following transition from study conditions [47]. However, previous experience shows that this may be unrealistic. The potential impacts that increased workloads will have on CHW motivation/retention if they are not remunerated were not addressed. Moreover, other potential macroeconomic benefits of extra malaria cases averted by the age-expanded mCCM were not accounted for, for example education outcomes or agricultural productivity [48], nor were the equity benefits of the expanded programme considered.

Age-expanded mCCM can significantly reduce the malaria burden in underserved rural areas by improving access to essential services and addressing healthcare inequities. The cost-effectiveness and risk aversion analysis provide strong evidence for policymakers, showing favourable economic value with minimal risk. Despite evidence gaps, these results highlight the need for national authorities to revisit mCCM policies, including care for all ages and implementing suitable compensation schemes to strengthen community-based healthcare delivery.

## Supplementary Information

Supplementary material 1

## Data Availability

The datasets supporting the conclusions of this article are included within the article (and its additional files). Analytic code used to generate the results in this article is available on github (https://github.com/wobiero/Madagascar-CEA/blob/main/Copy_of_mada_mccm.ipynb).

## Abbreviations

ACT: Artemisinin-based combination therapy
CE: Cost-effectiveness
CEAC: Cost-effectiveness acceptability curves
CEAFC: Cost-effectiveness affordability curves
CERAC: Cost-effectiveness risk-aversion curve
CET: Cost effectiveness analysis threshold
CHW: Community health workers
iCCM: Integrated community case management
ICER: Incremental cost-effectiveness ratios
DALY: Disability-Adjusted Life Year
EVPI: Expected value of perfect information
GDP: Gross domestic product
mCCM: Malaria community case management
PSA: Probabilistic sensitivity analyses
RDT: Rapid diagnostic tests
SSA: Sub-Saharan Africa
VSLY: Value of statistical life year
