# Supplementary material for: Bringing malaria diagnosis and treatment closer to the people: economic rationale for expanding malaria community case management to all ages in a rural district in Madagascar
Source: Malar J. 2025 May 4;24:141. doi: 10.1186/s12936-025-05381-y (PMC12049781; doi:10.1186/s12936-025-05381-y)
Supplement: Supplementary file 1 — Supplementary material 1 [file 12936_2025_5381_MOESM1_ESM.docx]

**TECHNICAL APPENDIX to Economic rationale for expanding malaria community case management to all ages in a rural district in Madagascar**

**Supplementary Table 1: Key assumptions for model parameters including values, distributions, and sources**

| Parameter | Value | Distribution | Parameters | Source |
| --- | --- | --- | --- | --- |
| Case fatality rate | 0.26% [0.15, 0.34] | Beta | α = 19.45  β = 59.56 | [1] [2] |
| Proportion severe hospitalized | .65[.50, .80] | Beta | α = 24.29  β = 12.78 | [1, 3] |
| Admission reduction | .84[.78, .90] | Beta | α = 121.64  β = 22.66 | Ashton et al (2023)[4] |
| Death reduction | .78[.68,.89] | Beta | α = 45.04  β = 12.01 | Ashton et al (2023)[4] |
| Duration uncomplicated malaria | 5.1[3.4, 7.3] | Log normal | μ = 1.61  σ = 0.20 | [3, 5] |
| Duration severe malaria | 19.7 [13.5, 27.9] | Log normal | μ = 2.96  σ = 0.20 | [3, 5, 6] |
| Length of stay | 5(3-16) | Log normal | μ = 2.33  σ = 0.20 | [1] |
| Length of stay with complications | 10.5[7.0, 15.1] | Log normal | μ = 2.32  σ = 0.27 | [1] |
| *Disability weights* | | | | |
| Uncomplicated Malaria | .051 [.032, 0.074] | Beta | α = 23.67  β = 440.52 | [7] |
| Severe malaria | .178 [.122, .247] | Beta | α = 20.37  β = 94.08 | [7] |
| Cerebral malaria | .471 | Beta | α = 20.23  β = 22.73 | [7] |
| Cerebral malaria and anemia | .620 | Beta | α = 8.88  β = 5.44 | [7] |
| DALYs per malaria death | 28.76[17.43, 44.88] | Gamma | α = 17.15  λ = 0.59 | Own calculations,[1] |
| *Complication risk* | | | | |
| Severe anemia | .322[.043, .77] | Beta | α = 16.63  β = 35.01 | [5, 7, 8] |
| Cerebral malaria | .002[.001, .003] | Beta | α = 24.95  β = 12449.05 | [5, 7] |
| Neurological deficits | .032[.020, .050] | Beta | α = 24.12  β = 731.08 | [7, 9] |
| Death with SMA | .097 | Point |  | [5-8] |
| Death with cerebral malaria | .192 | Point |  | [5, 7, 10] |
| Death with neurological sequelae | .192 | Point |  | [5, 9, 10] |
| Percentage transfused | 1% | Point |  | [1, 11] |
| *Economic parameters* | | | | |
| Exchange rate | 3905.4 Ariary to 1 USD | Point |  | IMF |
| Discount rate | 3% [0%-7%] | Point |  |  |
| Hourly wage nominal | $0.19 | Point |  | IMF |
| Hourly wage real | $0.75 | Point |  | IMF |
| GDP per capita | $516.59 | Point |  | IMF |
| GDP deflator 2022 | 281.07 | Point |  | IMF |
| GDP deflator 2010 | 126.68 | Point |  | IMF |
| Inflation rate | 6.0% | Point |  | IMF |
| Inequality adjustment | 0.057 | Point |  | IMF |
| ^1^OPD non-treatment costs 2022 | $1.49 [0.96, 2.17] | Gamma | α = 24.82  λ = 16.75 | WHO CHOICE (2010) |
| ^1^IPD non-treatment costs 2022 | $6.50 [4.23, 9.18] | Gamma | α = 26.24  λ = 4.05 | WHO CHOICE (2010) |
| Admission costs | $105.83 [77.36, 145.10] | Gamma | α = 38.29  λ = 0.36 | Own calculations |
| Wastage rate | 5% | Point |  | Actual data |
| Cost effectiveness threshold | $132.92 [$25.60, $690.2] | Uniform |  | [12, 13],Own calculations |
| *Population values* | | | | |
| Life expectancy Madagascar | 67.8 | Point |  |  |
| Number of CHWs Farafangana | 624 | Point |  | Own calculations [14] |
| Fever cases seen by CHWs per year | 387,624 | Point |  | Own calculations [14] |
| RDTs done by CHWs per year | 375,768 | Point |  | Own calculations [14] |
| ACTs given by CHWs per year | 257,899 | Point |  | Own calculations [14] |
| ^2^Incremental fever cases seen by CHWs | 183,207 | Gamma | α = 16.01  ^3^θ = 11,450.44 | Own calculations [14] |
| ^2^Incremental RDTs done by CHWs | 177,561 | Gamma | α = 11.11  ^3^θ = 15,980.49 | Own calculations [14] |
| ^2^Incremental ACTs given by CHWs | 122,889 | Gamma | α = 11.11  ^3^θ = 11,060.01 | Own calculations [14] |
| Admissions averted | 1,687.48 [169.90, 6,645.51] | Gamma | α = 30.29  λ = 0.01 | Own calculations [4] |
| Deaths averted | 99.70 [60.42, 155.55] | Gamma | α = 17.15  λ = 0.17 | Own calculations [4] |
| Simulations | 10,000 | Point |  | [15] |

1: Deflated values from WHO CHOICE 2010

2: Difference-in-difference estimates

3: Scale parameter -- θ shown instead of λ due to decimal points

**Supplementary Table 2. Comparison of population and health system characteristics in the study area by arm, 2019–2021**

|  | **Control** | **Intervention** | **Total** |
| --- | --- | --- | --- |
| **Population 2019** | | | |
| All Ages | 177 858 | 186 104 | 363 962 |
| 0-5 years | 40 372 | 42 245 | 82 617 |
| 6-13 years | 46 421 | 48 577 | 94 998 |
| 14+ years | 91 065 | 95 282 | 186 347 |
| **Health center level (01/2019 – 12/2021)** | | | |
| Number of facilities | 15 | 15 | 30 |
| Consultations | 239 679 | 222 536 | 462 215 |
| Fever cases | 160 367 | 147 385 | 307 752 |
| RDTs done | 193 294 | 188 893 | 382 187 |
| Malaria cases (RDT+) | 111 791 | 110 054 | 221 845 |
| ACTs administered | 108 708 | 108 494 | 217 202 |
| **Community health level (01/2019 – 12/2021)** | | | |
| Number of CHWs | 262 | 240 | 502 |
| Consultations | 201 555 | 262 885 | 464 440 |
| Fever cases | 177 709 | 244 562 | 422 271 |
| RDTs done | 154 785 | 215 482 | 370 267 |
| Malaria cases (RDT+) | 94 436 | 148 246 | 242 682 |
| ACTs administered | 87 282 | 141 022 | 228 304 |

**Supplementary Table 3: Potential compensation and the probability that the intervention was cost effective across different cost-effectiveness thresholds – health sector perspective.**

| Compensation | $26 per DALY averted | $133 per DALY averted | $258 per DALY averted | $517 per DALY averted | $690 per DALY averted |
| --- | --- | --- | --- | --- | --- |
| $1 | 6.70% | 57.30% | 98.30% | 100.00% | 100.00% |
| $10 | 5.60% | 49.00% | 97.20% | 100.00% | 100.00% |
| $20 | 4.70% | 40.40% | 95.70% | 100.00% | 100.00% |
| $30 | 4.00% | 32.10% | 93.00% | 100.00% | 100.00% |
| $40 | 3.4% | 25.60% | 89.60% | 100.00% | 100.00% |
| $50 | 2.90% | 20.30% | 84.70% | 100.00% | 100.00% |
| $60 | 2.50% | 15.80% | 79.70% | 100.00% | 100.00% |
| $70 | 2.20% | 12.30% | 73.70% | 100.00% | 100.00% |
| $80 | 2.00% | 9.70% | 67.30% | 99.90% | 100.00% |
| $90 | 1.60% | 7.40% | 60.40% | 99.80% | 100.00% |
| $100 | 1.40% | 6.10% | 53.80% | 99.50% | 100.00% |

**Supplementary Table 4: Probability cost effective by monthly CHW compensation and cost-effectiveness threshold – health sector perspective**

|  | Compensation | CET_$26 | CET_$50 | CET_$100 | CET_$133 | CET_$150 | CET_$200 | CET_$250 | CET_$300 | CET_$350 | CET_$400 | CET_$450 | CET_$500 | CET_$550 | CET_$600 | CET_$650 |
| --- | --- | --- | --- | --- | --- | --- | --- | --- | --- | --- | --- | --- | --- | --- | --- | --- |
| 0 | $1.00 | 81.40% | 93.60% | 99.70% | 100.00% | 100.00% | 100.00% | 100.00% | 100.00% | 100.00% | 100.00% | 100.00% | 100.00% | 100.00% | 100.00% | 100.00% |
| 1 | $5.00 | 74.00% | 89.70% | 99.30% | 99.90% | 100.00% | 100.00% | 100.00% | 100.00% | 100.00% | 100.00% | 100.00% | 100.00% | 100.00% | 100.00% | 100.00% |
| 2 | $10.00 | 63.60% | 82.80% | 98.30% | 99.80% | 99.90% | 100.00% | 100.00% | 100.00% | 100.00% | 100.00% | 100.00% | 100.00% | 100.00% | 100.00% | 100.00% |
| 3 | $15.00 | 53.40% | 73.50% | 96.60% | 99.30% | 99.80% | 100.00% | 100.00% | 100.00% | 100.00% | 100.00% | 100.00% | 100.00% | 100.00% | 100.00% | 100.00% |
| 4 | $20.00 | 43.80% | 63.80% | 93.40% | 98.50% | 99.30% | 99.90% | 100.00% | 100.00% | 100.00% | 100.00% | 100.00% | 100.00% | 100.00% | 100.00% | 100.00% |
| 5 | $25.00 | 35.60% | 53.90% | 88.80% | 97.10% | 98.60% | 99.90% | 100.00% | 100.00% | 100.00% | 100.00% | 100.00% | 100.00% | 100.00% | 100.00% | 100.00% |
| 6 | $30.00 | 29.00% | 44.80% | 82.00% | 94.90% | 97.40% | 99.70% | 100.00% | 100.00% | 100.00% | 100.00% | 100.00% | 100.00% | 100.00% | 100.00% | 100.00% |
| 7 | $35.00 | 23.30% | 36.60% | 74.10% | 90.80% | 95.30% | 99.40% | 99.90% | 100.00% | 100.00% | 100.00% | 100.00% | 100.00% | 100.00% | 100.00% | 100.00% |
| 8 | $40.00 | 19.00% | 29.60% | 65.40% | 85.30% | 91.60% | 98.70% | 99.90% | 100.00% | 100.00% | 100.00% | 100.00% | 100.00% | 100.00% | 100.00% | 100.00% |
| 9 | $45.00 | 16.10% | 24.40% | 56.50% | 78.50% | 86.80% | 97.50% | 99.70% | 99.90% | 100.00% | 100.00% | 100.00% | 100.00% | 100.00% | 100.00% | 100.00% |
| 10 | $50.00 | 13.50% | 19.80% | 47.90% | 71.10% | 80.80% | 95.90% | 99.30% | 99.90% | 100.00% | 100.00% | 100.00% | 100.00% | 100.00% | 100.00% | 100.00% |
| 11 | $55.00 | 11.40% | 16.50% | 40.30% | 62.90% | 73.60% | 93.50% | 98.70% | 99.80% | 100.00% | 100.00% | 100.00% | 100.00% | 100.00% | 100.00% | 100.00% |
| 12 | $60.00 | 9.70% | 13.90% | 33.50% | 54.60% | 66.40% | 89.50% | 97.60% | 99.60% | 99.90% | 100.00% | 100.00% | 100.00% | 100.00% | 100.00% | 100.00% |
| 13 | $65.00 | 8.40% | 11.70% | 27.30% | 46.60% | 58.00% | 84.80% | 96.10% | 99.30% | 99.90% | 100.00% | 100.00% | 100.00% | 100.00% | 100.00% | 100.00% |
| 14 | $70.00 | 7.30% | 9.90% | 22.40% | 39.70% | 50.10% | 79.20% | 94.20% | 98.60% | 99.70% | 99.90% | 100.00% | 100.00% | 100.00% | 100.00% | 100.00% |
| 15 | $75.00 | 6.40% | 8.60% | 18.50% | 33.70% | 43.20% | 73.00% | 91.20% | 97.60% | 99.50% | 99.90% | 100.00% | 100.00% | 100.00% | 100.00% | 100.00% |
| 16 | $80.00 | 5.70% | 7.60% | 15.70% | 27.80% | 36.90% | 66.40% | 87.50% | 96.30% | 99.20% | 99.80% | 99.90% | 100.00% | 100.00% | 100.00% | 100.00% |
| 17 | $85.00 | 5.00% | 6.50% | 13.00% | 23.00% | 31.30% | 59.20% | 82.80% | 94.40% | 98.50% | 99.60% | 99.90% | 100.00% | 100.00% | 100.00% | 100.00% |
| 18 | $90.00 | 4.40% | 5.70% | 11.20% | 19.20% | 26.00% | 52.10% | 77.80% | 92.10% | 97.60% | 99.40% | 99.90% | 100.00% | 100.00% | 100.00% | 100.00% |
| 19 | $95.00 | 4.00% | 5.10% | 9.50% | 16.10% | 21.50% | 45.40% | 72.00% | 89.30% | 96.50% | 99.00% | 99.70% | 99.90% | 100.00% | 100.00% | 100.00% |
| 20 | $100.00 | 3.50% | 4.50% | 8.20% | 13.60% | 18.20% | 39.70% | 66.00% | 85.40% | 94.60% | 98.40% | 99.60% | 99.90% | 100.00% | 100.00% | 100.00% |
| 21 | $105.00 | 3.30% | 4.00% | 7.20% | 11.80% | 15.20% | 34.40% | 59.80% | 80.90% | 92.70% | 97.60% | 99.30% | 99.80% | 99.90% | 100.00% | 100.00% |
| 22 | $110.00 | 3.00% | 3.60% | 6.20% | 10.00% | 13.00% | 29.40% | 53.60% | 76.40% | 90.30% | 96.50% | 98.80% | 99.60% | 99.90% | 100.00% | 100.00% |
| 23 | $115.00 | 2.60% | 3.30% | 5.60% | 8.60% | 11.20% | 25.00% | 47.50% | 71.40% | 87.40% | 94.80% | 98.40% | 99.50% | 99.80% | 99.90% | 100.00% |
| 24 | $120.00 | 2.40% | 3.00% | 4.90% | 7.30% | 9.60% | 21.10% | 42.00% | 65.70% | 83.50% | 93.00% | 97.60% | 99.20% | 99.70% | 99.90% | 100.00% |
| 25 | $125.00 | 2.20% | 2.70% | 4.40% | 6.40% | 8.10% | 18.00% | 37.00% | 60.30% | 79.40% | 91.00% | 96.50% | 98.70% | 99.50% | 99.80% | 100.00% |
| 26 | $130.00 | 2.00% | 2.40% | 3.90% | 5.60% | 7.00% | 15.60% | 32.40% | 54.90% | 75.00% | 88.60% | 95.00% | 98.30% | 99.40% | 99.80% | 99.90% |
| 27 | $135.00 | 1.70% | 2.20% | 3.40% | 5.00% | 6.20% | 13.20% | 28.20% | 49.10% | 70.30% | 85.50% | 93.20% | 97.60% | 99.00% | 99.60% | 99.90% |
| 28 | $140.00 | 1.60% | 2.00% | 3.20% | 4.40% | 5.40% | 11.40% | 24.40% | 44.10% | 65.20% | 81.80% | 91.40% | 96.40% | 98.60% | 99.50% | 99.80% |
| 29 | $145.00 | 1.40% | 1.80% | 2.80% | 3.90% | 4.80% | 9.70% | 21.20% | 39.30% | 60.50% | 77.70% | 89.40% | 95.00% | 98.20% | 99.30% | 99.70% |
| 30 | $150.00 | 1.30% | 1.60% | 2.60% | 3.40% | 4.20% | 8.20% | 18.30% | 34.80% | 55.50% | 73.90% | 86.80% | 93.40% | 97.40% | 98.90% | 99.50% |

**Supplementary Figures**

**Supplementary Figure 1: Decision model for the impact of age-expanded mCCM in Madagascar.**

CHW – community health worker; OPD – outpatient department; IP – inpatient; Rx – treatment; and other – treatment outside the formal health system. The structure at the top for early presentation to CHW care is replicated at the bottom blue node for late presentation to CHW care. The CHW model structure is replicated for OPD care – red node.

*
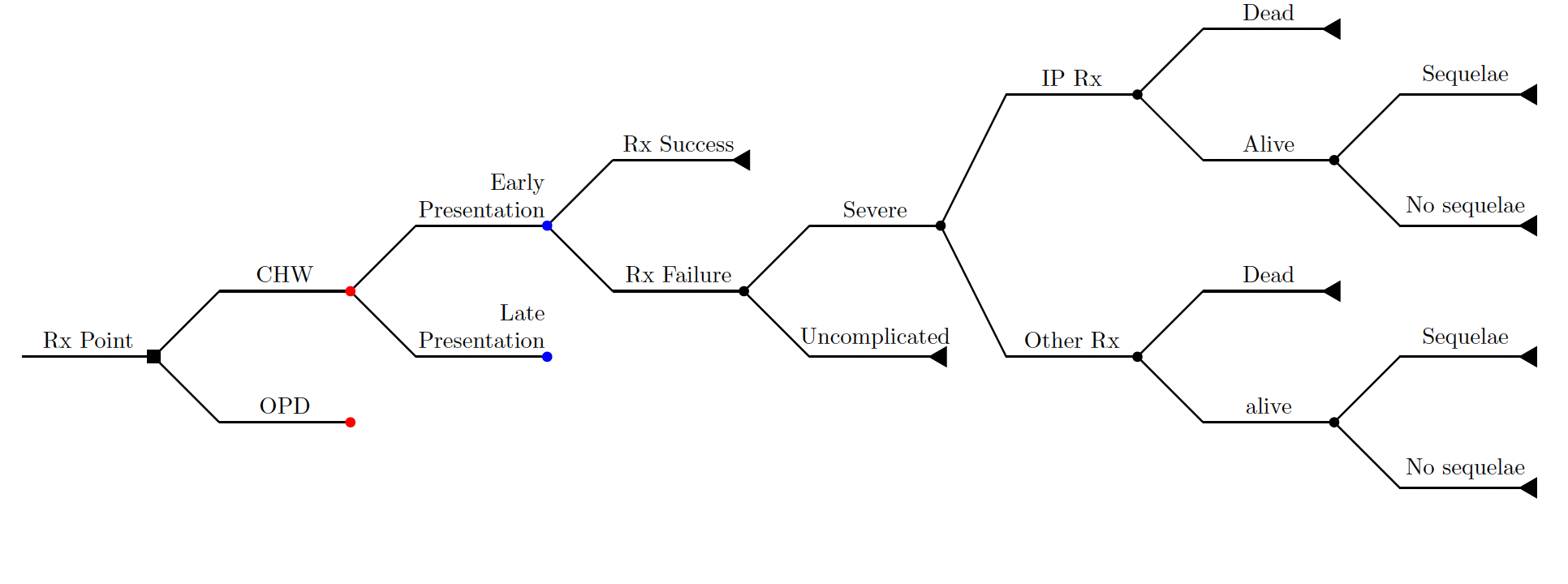
*

*Inpatient costs*

The inpatient hoteling cost estimates are derived from WHO CHOICE estimates adjusted using a GDP deflator. These WHO CHOICE estimates encompass personnel, capital and operational costs but do not include consumables and drugs. We assume that all severe patients are treated with three doses of parenteral artesunate, followed by oral artemether-lumefantrine (AL). We assume 30% of patients require additional IV artesunate doses. Each patient receives three intravenous (IV) infusions (Dextrose, Ringers), but only one IV set is used. Five percent of the patients receive blood transfusions. Additionally, all patients receive a full hemogram (full blood counts), with one quarter also receiving microscopy. Nurses take vital signs twice per shift throughout the admission period for consumable estimates, and no additional feeding, such as nasogastric tubes, is provided. Nurses administer IV artesunate separately from vital signs. Furthermore, it is assumed that 25% of the patients receive oral ferrous sulphate (FeSO4) for three months post-discharge.

**Supplementary Figure 2: Interpretation of cost-effectiveness affordability curves**

The cost effectiveness plane below shows an analysis done from a health system perspective and assuming a monthly compensation of $63 per month for CHWs and a CET of $133 per DALY averted – blue dotted line (λ). Based on 10,000 Monte Carlo simulations, there is a 45% probability that the intervention is cost-effective. That is, 45% of the posterior joint distribution of incremental costs and incremental effects falls to the right and below the CET[16].

The x-axis represents the budgetary status quo. Changes to the budget are represented by vertical shifts (red dotted lines) labelled ω_1_ – increase, and ω_2_ – decrease.

Using a budgetary increase -- ω_1_ -- for illustration:

- Zone I - represents a region where the intervention is cost-effective, but financially infeasible as it lies above the increased budget line.
- Zone II – represents a region where the intervention is both cost-effective and feasible given the increased resources.
- Zone III – represents a region where the intervention is financially feasible but is not cost-effective.
- Zone IV – represents a region where the intervention is neither cost-effective nor financially feasible.

By shifting the budget line up or down along the y-axis, while keeping the CE threshold constant, the probability that the intervention is both cost-effective and financially feasible can be assessed[16]. In the current simulations, there is a 29% probability the intervention is both feasible and cost-effective assuming a $375K budgetary increase. By shifting budget lines and cost effectiveness thresholds, cost-effectiveness acceptability curves can be created[16, 17]. In case of budget cuts ω_2_, the new intervention may be infeasible even if cost-effective[16, 17].


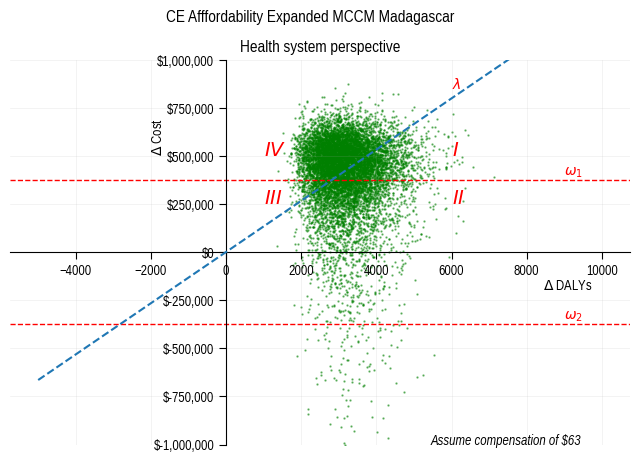


**Supplementary Figure 3: Cost-effectiveness analysis plane from a health sector perspective comparing the impact of nominal compensation for CHWs versus $100 per month.**


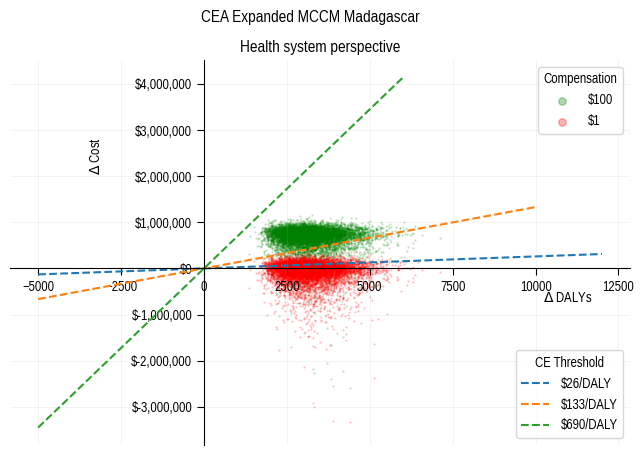


**Supplementary Figure 4: Net monetary benefit of age-expanded MCCM from a health system perspective.** Figure shows the link between the potential economic benefits with choice of cost-effectiveness threshold.


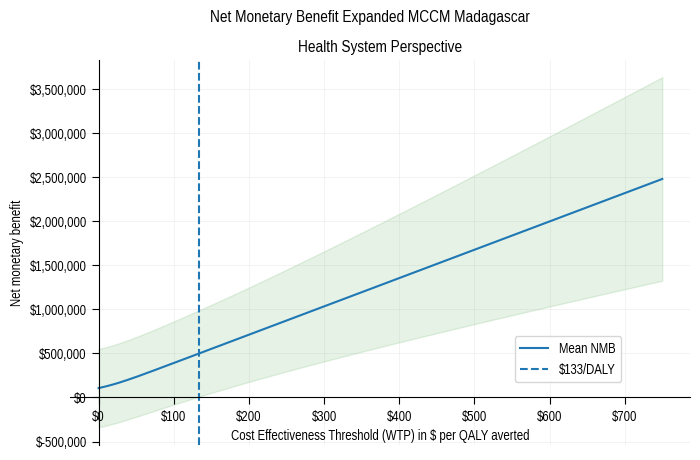


**Supplementary Figure 5: The cost-effectiveness risk aversion curve showing the net-benefit-to-risk ratio of the age-expanded mCCM project from a health system perspective.** There is minimal downside risk of investing in the project above a CET of $186 per DALY averted.


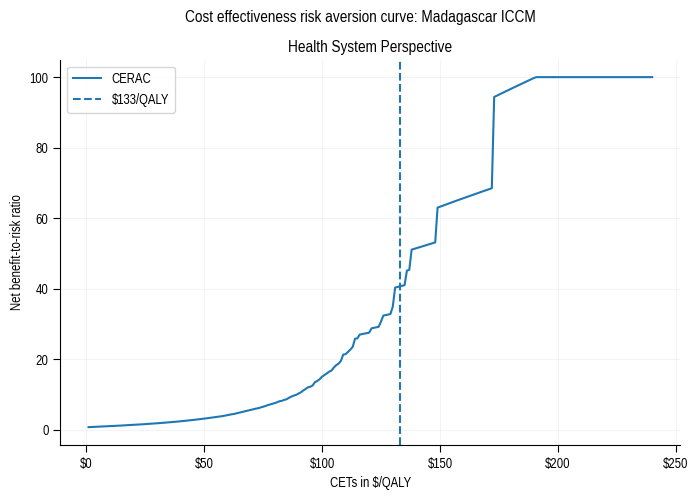


**Supplementary Figure 6: Univariate sensitivity analysis from a societal perspective with potential averted death benefits included.**
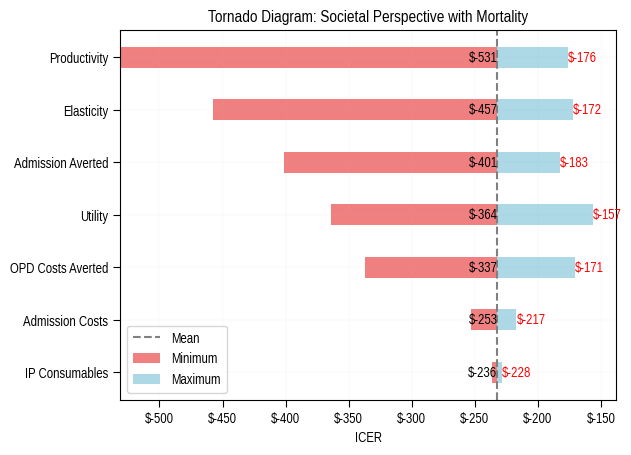


**Supplementary Figure 7: Impact of elasticity estimates used for calculating the value of statistical life year on cost-effectiveness**


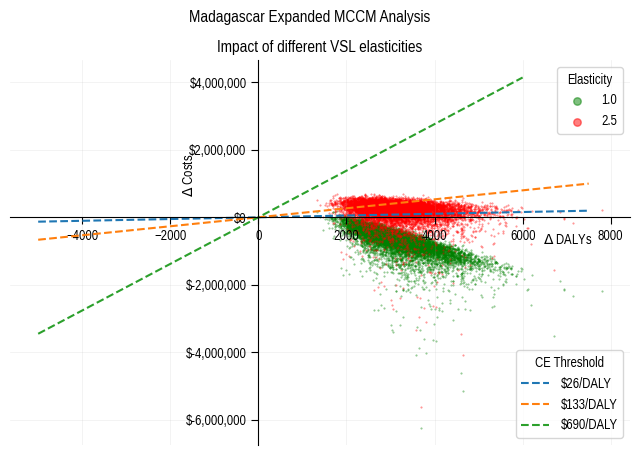


**References**

1. Alonso S, Chaccour CJ, Elobolobo E, Nacima A, Candrinho B, Saifodine A, et al. The economic burden of malaria on households and the health system in a high transmission district of Mozambique. Malar J. 2019;18(1):360. Epub 2019/11/13. doi: 10.1186/s12936-019-2995-4. PubMed PMID: 31711489; PubMed Central PMCID: PMCPMC6849240.

2. Hay SI, Snow RW. The malaria Atlas Project: developing global maps of malaria risk. PLoS Med. 2006;3(12):e473. Epub 2006/12/07. doi: 10.1371/journal.pmed.0030473. PubMed PMID: 17147467; PubMed Central PMCID: PMCPMC1762059.

3. Mousa A, Al-Taiar A, Anstey NM, Badaut C, Barber BE, Bassat Q, et al. The impact of delayed treatment of uncomplicated P. falciparum malaria on progression to severe malaria: A systematic review and a pooled multicentre individual-patient meta-analysis. PLoS Med. 2020;17(10):e1003359. Epub 2020/10/20. doi: 10.1371/journal.pmed.1003359. PubMed PMID: 33075101; PubMed Central PMCID: PMCPMC7571702 following competing interests: PH works for Medicines for Malaria Venture (MMV), which has a Research Collaboration Agreement in place with Imperial College. LCO declares grant funding from the World Health Organization, the Bill and Melinda Gates Foundation, and Medicines for Malaria Venture.

4. Ashton RA, Hamainza B, Lungu C, Rutagwera MI, Porter T, Bennett A, et al. Effectiveness of community case management of malaria on severe malaria and inpatient malaria deaths in Zambia: a dose-response study using routine health information system data. Malar J. 2023;22(1):96. Epub 2023/03/18. doi: 10.1186/s12936-023-04525-2. PubMed PMID: 36927440; PubMed Central PMCID: PMCPMC10022244.

5. Greenhalgh S, Chandwani V. Advocating an attack against severe malaria: a cost-effectiveness analysis. BMC Public Health. 2020;20(1):17. Epub 2020/01/09. doi: 10.1186/s12889-019-8141-y. PubMed PMID: 31910842; PubMed Central PMCID: PMCPMC6947859.

6. Winskill P, Dhabangi A, Kwambai TK, Mori AT, Mousa A, Okell LC. Estimating the burden of severe malarial anaemia and access to hospital care in East Africa. Nat Commun. 2023;14(1):5691. Epub 2023/09/15. doi: 10.1038/s41467-023-41275-w. PubMed PMID: 37709763; PubMed Central PMCID: PMCPMC10502125.

7. Network GBoDC. Global Burden of Disease Study 2019 (GBD 2019). 2020.

8. White NJ. Anaemia and malaria. Malar J. 2018;17(1):371. Epub 2018/10/21. doi: 10.1186/s12936-018-2509-9. PubMed PMID: 30340592; PubMed Central PMCID: PMCPMC6194647.

9. Brewster DR, Kwiatkowski D, White NJ. Neurological sequelae of cerebral malaria in children. Lancet. 1990;336(8722):1039-43. Epub 1990/10/27. doi: 10.1016/0140-6736(90)92498-7. PubMed PMID: 1977027.

10. Idro R, Marsh K, John CC, Newton CR. Cerebral malaria: mechanisms of brain injury and strategies for improved neurocognitive outcome. Pediatr Res. 2010;68(4):267-74. Epub 2010/07/08. doi: 10.1203/PDR.0b013e3181eee738. PubMed PMID: 20606600; PubMed Central PMCID: PMCPMC3056312.

11. Avancena ALV, Miller A, Canana N, Dula J, Saifodine A, Cadrinho B, et al. Achieving malaria testing and treatment targets for children under five in Mozambique: a cost-effectiveness analysis. Malar J. 2022;21(1):320. Epub 2022/11/08. doi: 10.1186/s12936-022-04354-9. PubMed PMID: 36344998; PubMed Central PMCID: PMCPMC9641811.

12. Beth Woods PR, Mark Sculpher, Karl Claxton. Country-level cost effectiveness thresholds: Initial estimates and the need for further research. Value in Health. 2016;(19):926-35.

13. Viscusi WK, Huber J, Bell J. Assessing whether there is a cancer premium for the value of a statistical life. Health Econ. 2014;23(4):384-96. Epub 2013/03/23. doi: 10.1002/hec.2919. PubMed PMID: 23520055.

14. Garchitorena A, Harimanana A, Irinantenaina J, Razanadranaivo HL, Rasoanaivo TF, Sayre D, et al. Expanding community case management of malaria to all ages can improve universal access to malaria diagnosis and treatment: results from a cluster randomized trial in Madagascar. BMC Med. 2024;22(1):231. Epub 2024/06/10. doi: 10.1186/s12916-024-03441-9. PubMed PMID: 38853263; PubMed Central PMCID: PMCPMC11163690.

15. Jiahang Zhang SC. Investigating the Number of Monte Carlo Simulations for Statistically Stationary Model Outputs. Axioms. 2023;12:481.

16. Sendi P, Matter-Walstra K, Schwenkglenks M. Handling Uncertainty in Cost-Effectiveness Analysis: Budget Impact and Risk Aversion. Healthcare (Basel). 2021;9(11). Epub 2021/11/28. doi: 10.3390/healthcare9111419. PubMed PMID: 34828466; PubMed Central PMCID: PMCPMC8622052.

17. Sendi PP, Briggs AH. Affordability and cost-effectiveness: decision-making on the cost-effectiveness plane. Health Econ. 2001;10(7):675-80. Epub 2001/12/18. doi: 10.1002/hec.639. PubMed PMID: 11747050.
